# Electrophysiological correlates of gist perception: a steady-state visually evoked potentials study

**DOI:** 10.1007/s00221-020-05819-6

**Published:** 2020-05-03

**Authors:** Elise L. Radtke, Benjamin Schöne, Ulla Martens, Thomas Gruber

**Affiliations:** 1 Institute of Psychology, Osnabrück University, Seminarstraße 20, 49074 Osnabrück, Germany; 2 DRK-Norddeutsches Epilepsiezentrum für Kinder und Jugendliche, Henry-Dunant-Str. 6-10, 24223 Schwentinental, Germany

**Keywords:** EEG, Steady-state visually evoked potentials, Intermodulation frequency, Multi-stimulus displays, Gist perception

## Abstract

Gist perception refers to perceiving the substance or general meaning of a scene. To investigate its neuronal mechanisms, we used the steady-state visually evoked potential (SSVEP) method—an evoked oscillatory cortical response at the same frequency as a visual stimulus flickered at this frequency. Two neighboring stimuli were flickered at different frequencies *f*_*1*_ and *f*_*2*_, for example, a drawing of a sun on the left side of the screen flickering at 8.6 Hz and the drawing of a parasol on the right side of the screen flickering at 12 Hz. SSVEPs enabled us to separate the responses to the two distinct stimuli by extracting oscillatory brain responses at *f*_*1*_ and *f*_*2*_. Additionally, it allowed to investigate intermodulation frequencies, that is, the brain’s response at a linear combination of *f*_*1*_ and *f*_*2*_ (here at *f*_*1*_ + *f*_*2*_ = 20.6 Hz) as an indicator of processing shared aspects of the input, that is, gist perception (here: a beach scene). We recorded high-density EEG of 18 participants. Results revealed clear and separable neuronal oscillations at *f*_*1*_ and *f*_*2*_. Additionally, occipital electrodes showed increased amplitudes at the intermodulation frequency in related as compared to unrelated pairs. The increase in intermodulation frequency was associated with bilateral temporal and parietal lobe activation, probably reflecting the interaction of local object representations as a basis for activating the gist network. The study demonstrates that SSVEPs are an excellent method to unravel mechanisms underlying the processing within multi-stimulus displays in the context of gist perception.

## Introduction

Encounters with an object under natural circumstances unlikely occur under isolated conditions. Rather, many objects co-occur within a scene (e.g., the sun and a parasol). While object recognition (e.g., Singer 1995; Tanaka 1993) and scene perception (e.g., Henderson and Hollingworth 1999) are widely studied separately, the underlying neuronal processes of gist perception are not understood to their full extent. Gist perception, that is, grasping the meaning of a scene at a single glance, relies on the automatic activation of semantic information (Oliva 2005) and integrating the separate objects into a coherent scene (Bar 2004). Therefore, gist perception can be perfectly investigated by inducing semantic relatedness on multi-stimulus displays by presenting a background and figure or by presenting semantically related objects. In an evoked gamma-band study (i.e., event-related oscillations around 40 Hz), it was shown that related object pairs revealed significantly larger evoked gamma-band responses as opposed to unrelated objects already 70–130 ms after stimulus onset (Oppermann et al. 2012).

The present study aimed to further examine multi-stimulus processing and gist perceptions by means of electroencephalography (EEG). In particular, we applied steady-state visual evoked potentials (SSVEP), that is, an oscillatory cortical response at the same frequency as a visual stimulus flickered at this frequency (Regan 1989). In contrast to conventional neuroscientific methods (e.g., the BOLD response or event-related potentials) that reflect the total signal elicited by all components of a multi-stimulus display, the SSVEP can be used to separate brain responses of the objects constituting the complete visual input. In the example given above, the sun and the parasol could be presented simultaneously, tagged with different frequencies. The sun might flicker at *f*_1_ = 8.57 Hz and the parasol at *f*_2_ = 12 Hz. This multi-stimulus display will elicit two SSVEPs at the respective driving frequencies at *f*_1_ and *f*_2_. By means of a spectral decomposition of the EEG data, the *f*_1_- and *f*_2_-related brain oscillations can now be examined simultaneously.

In general, SSVEP studies have revealed that amplitude modulations at the driving frequencies reflect attentional processing (Hillyard et al. 1997; Morgan et al. 1996; Müller et al. 2003; Müller and Hillyard 2000; for a review see Vialatte et al. 2010), memory (Martens et al. 2012; Silberstein et al. 2001) and object recognition (Kaspar et al. 2010). The application of the SSVEP approach in multi-stimulus paradigms is suitable to measure brain activity related specifically to each stimulus separately. For example, activity of lateral cortex regions was related to the processing of figures, whereas a separate network extending from visual cortices V1, V2, and V3, through more dorsal areas was related to the processing of background information (Appelbaum et al. 2006). Another study showed that background processing is increased in consistent scenes, whereas object-related processing is increased in inconsistent scenes—suggesting that inconsistency is associated with an attention focus on the object, whereas consistency is associated with an attention focus on the background (Martens et al. 2011). Besides the above influences, the SSVEP is also affected by low-level stimulus properties such as contrast and luminance (Vialatte et al. 2010; Wieser et al. 2016). This calls for a thorough control of these features, which we applied in our study.

More recently, a complementary approach to SSVEP analyses has been used. When frequency-tagging multiple stimuli, additionally to the oscillatory brain response’s changes at the driving frequencies *f*_1_ and *f*_2_, peaks occur at the so-called intermodulation frequencies, that is, at sums and differences of integer multiplies of the driving frequencies, for example, *f*_1_ + *f*_2_ = 20.57 Hz or 2 × (*f*_1_ + *f*_2_) = 41.14 Hz. They are generated by common nonlinear processing of the input (Ratliff and Zemon 1980; Regan and Regan 1988; Zemon and Ratliff 1982, 1984).

In multi-stimulus SSVEP studies, intermodulation frequencies were found to be sensitive to visual integration and perceptual binding. In a study by Gundlach and Müller (2013), two differently flickering stimuli were presented and the formation of an illusory rectangle occurred. The brain increasingly oscillated at the intermodulation frequency when the illusory figure was perceived compared to not perceiving it. Similarly, when moving bars are presented at two frequencies, intermodulation power was more increased during perceptual form or motion integration than during perceiving the stimuli as segmented components moving individually (Aissani et al. 2011). Furthermore, intermodulation frequencies indicate higher order binding: When presenting two face halves at different frequencies, activation was found to increase at the intermodulation components over the right occipito-temporal hemisphere in cases in which a complete face was perceived (Boremanse et al. 2013).

To sum, the application of the SSVEP technique in multiple stimulus displays in combination with the analyses of intermodulation frequencies makes this an ideal approach to investigate gist perception in multi-stimulus processing. In particular, we intended to examine if intermodulation frequencies are a suitable marker for the integration of semantically related objects, or in other words, a marker of gist perception. To that end, we presented frequency-tagged pairs of objects and manipulated their semantical relatedness. As a marker for processing the separate objects versus integrating them into a coherent scene, we expected different amplitude modulations of the driving versus intermodulation frequency when viewing related versus unrelated stimulus pairs, respectively. To examine the location of the cortical generators of gist perception, we modeled the SSVEP sources by means of a distributed source model variable resolution electromagnetic tomography (VARETA; Bosch-Bayard et al. 2001).

## Materials and methods

### Participants

Twenty-one students from Osnabrück University gave their informed consent and participated in the study. They all had normal or corrected to normal vision and no psychological or neurological disorder, specifically they had no migraine or epilepsy and took no medication. The study was approved by the Ethics Commitee of Osnabrück University.

Two participants were excluded due to technical problems during the recording and one participant due to uncorrectable EEG artifacts. The EEG data of the remaining 18 participants were used for further analysis. The behavioral data from one participant were accidentally lost, thus, the behavioral data of only 17 participants could be analyzed.

### Stimulus presentation

**Fig. 1:**
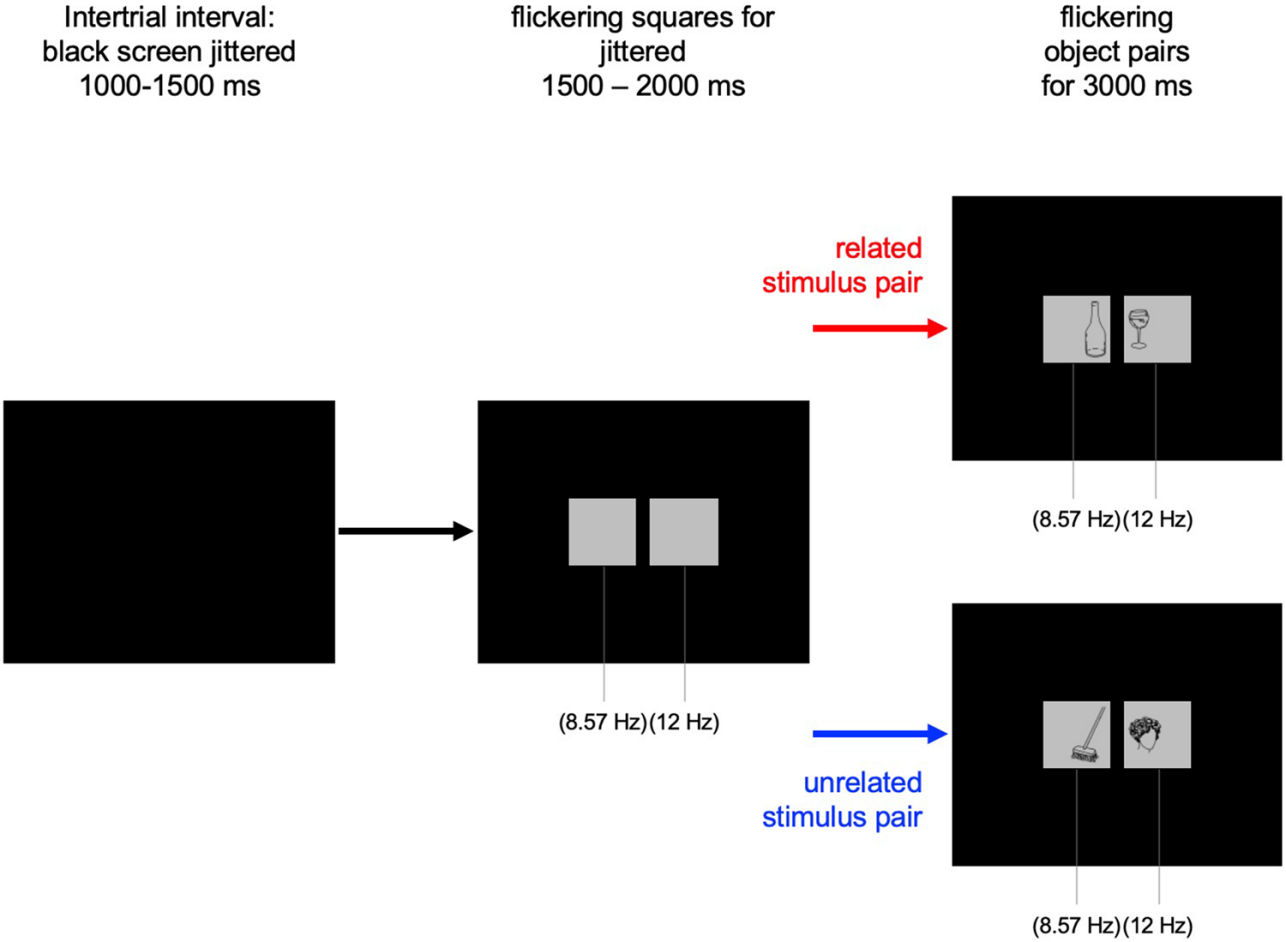
Trial procedure

We used 160 line drawings of objects superimposed on a gray background that were arranged in 80 pairings of coherent scenes (sharing a general meaning, e.g., a sun and a parasol, see Fig. 1 for an example). Forty pairings of related stimuli were assigned to Set 1 and 40 pairings of related stimuli to Set 2. The 40 related pairings from Set 1 were then rearranged to result in unrelated stimulus pairs, which were assigned to Set 2. The 40 related pairings from Set 2 were rearranged to result in unrelated pairings, which were assigned to Set 1. As a result, Set 1 and Set 2 each consisted of 40 related pairings and 40 unrelated pairings. During the experiment, half of the participants were confronted with stimuli from Set 1, to the other half, we presented Set 2. This assured that each object was used in a related context and an unrelated context, respectively, in a counterbalanced manner. We used flicker frequencies of *f*_1_ = 8.57 Hz and *f*_2_ = 12 Hz. These frequencies were used in previous studies (e.g., Martens et al. 2011), because the SSVEP signal is largest around 10 Hz (Herrmann 2001) and in this configuration *f*_*2*_ is not a harmonic (i.e., multiple) frequency of *f*_*1*_, which should be avoided when investigating intermodulation frequencies. In a first block, all 80 pairs of a set were presented—in 20 of the related trials and 20 of the unrelated trials, the left object was flickered at *f*_1_ (e.g., 8.57 Hz) and the right object at *f*_2_ (e.g., 12 Hz). For the other 40 pairs flickering was vice versa. In a second block, the same 80 pairs were presented, but flickering was reversed, compared to the first block. This assured that each object was seen in each of the two flicker frequencies. To present the stimuli, we used a monitor with a refresh rate of 60 Hz. The drawings were presented every 5th (12 Hz, with a duty cycle 1:4) or every 7th refresh cycle (8.57 Hz with a duty cycle of 1:6). The stimuli subtended a horizontal visual angle of 7.4° and a vertical visual angle of 3.4°, so that they were within parafoveal view of approx. 9° diameter (Strasburger et al. 2011). To ensure precise timing, we used Matlab Version 2015 and the Psychophysics Toolbox extensions (Brainard 1997).

### Stimulus characteristics

We calculated the fast Fourier transform for each object drawing, which results in a spectrogram containing information about the horizontal and vertical distribution of spatial frequencies. This spectrogram was subjected to an entropy calculation as a measure of randomness/complexity of the spectrogram. Second, we calculated the luminance, that is, the percentage of gray pixels, for each object drawing. To assure comparable stimulus properties, we conducted four 2 (related vs. unrelated context) × 2 (presented at left vs. right position) ANOVAs, one with luminance as the dependent variable and a second one with entropy as the dependent variable, for each stimulus set. This showed that there was no difference in entropy of the spatial information or luminance between any of the conditions or groups (Table 1).

**Table 1:** Stimuli characteristics. Did not differ between conditions, positions on the screen, and participant groups

	Relatedness effect						Position effect						Interaction effect	
	Related		Unrelated		Main Effect		Left Object		Right Object		Main Effect			
	*M*	(SD)	*M*	(SD)	*F*(1,39)	*p*	*M*	(SD)	*M*	(SD)	*F*(1,39)	*p*	*F*(1,39)	*p*
Luminance in % gray pixels (ranging from 0.70–13.00)														
Set 1	2.95	(1.77)	3.30	(1.99)	1.62	0.21	3.20	(1.63)	3.04	(2.10)	0.19	0.66	0.16	0.70
Set 2	3.30	(1.99)	2.95	(1.75)	1.66	0.21	3.25	(1.74)	2.99	(2.01)	0.55	0.46	0.31	0.58
Entropy (ranging from 0.003–3.50)														
Set 1	0.07	(0.05)	0.06	(0.06)	0.43	0.52	0.07	(0.06)	0.06	(0.05)	0.17	0.69	2.30	0.14
Set 2	0.06	(0.06)	0.07	(0.05)	0.29	0.60	0.06	(0.06)	0.07	(0.05)	0.48	0.49	1.00	0.32

Half the participants saw Set 1 and the other half saw Set 2

### Procedure

An exemplary time course of one experimental trial is depicted in Fig. 1. Each trial started with a blank screen for 1000–1500 ms, followed by two flickering gray squares for 1500–2000 ms in each visual field. These flickering squares served as a baseline period and they flickered at the same frequency as the upcoming superimposed line drawing. The line drawings, that is, related or unrelated object pairs, were presented for 3000 ms. Specifically, the stimuli in the left visual field were presented at a driving frequency of *f*_1_ = 8.57 Hz and in the right visual field at a driving frequency of *f*_2_ = 12 Hz, or vice versa.

In 24 of 160 trials, balanced across related and unrelated line drawings, a magenta dot was briefly (67 ms) superimposed on the objects. This target appeared between 100 and 2700 ms after the stimulus onset at a random position. Participants were asked to detect and report seeing the magenta dot by a button press. This task was introduced to uphold the participant’s attention towards the object pairs during the whole stimulation period. The EEG during target detection trials was not further analyzed.

### Electrophysiological recording

EEG was recorded using 128 electrodes and a BioSemi Active Two amplification system with a sampling rate of 512 Hz. Two additional electrodes were used as reference and ground (CMS and DRL; for more info see https://www.biosemi.com/faq/cms&drl.htm). Eye movements and blinks were measured by the vertical and horizontal electro-oculogram. For preprocessing and EEG analysis, we used Matlab Version 2015 and the EEGLab toolbox version 14 (Delorme and Makeig 2004).

The data was segmented into epochs from − 500 to 2900 ms relative to stimulus onset with a baseline from − 500 to 0 ms. Artifact correction was performed offline by means of the Fully Automated Statistical Thresholding for EEG artifact Rejection (FASTER; Nolan et al. 2010). FASTER is an automated and unsupervised approach comprising several steps. In particular, (1) channels with amplitude z-scores > 3 were interpolated, that is, the data from one experimental epoch at a specific channel are interpolated, if the averaged amplitude within this time series exceeds a z-score of 3 (in relation to all experimental epochs), (2) epochs with amplitude z-scores > 3 were removed, (3) the data was re-referenced to the average amplitude of all electrodes, (4) Independent Component Analysis (ICA) is performed, (5) ICA components with z-scores > 3 were rejected from the data and (6) finally channels within the remaining epochs with amplitude z-scores > 3 were interpolated. In the data of the 18 participants that were finally analyzed, on average per participant, 4.61 epochs (SD = 1.80, range = 1–8) out of 136 epochs and 7.61 components (SD = 2.19, range = 4–11) were rejected and 4.00 channels (SD = 2.13, range = 1–9) were rejected and interpolated. The number of remaining epochs per participant did not differ in congruent (*M* = 66.06, *SD* = 1.39) versus incongruent (*M* = 65.17, *SD* = 2.04) trials, *t*(17) = 1.28, *p* = 0.22.

**Fig. 2:**
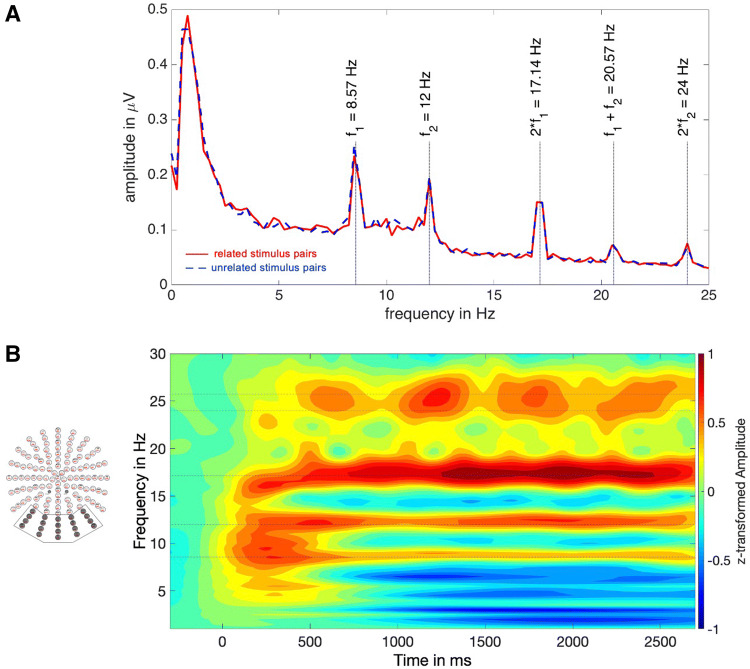
EEG amplitude spectra. **a** EEG amplitude spectrum averaged across all electrodes at − 1000 to 2900 ms. Only the flickered frequencies (*f*_1_ and *f*_2_), their harmonics (2 ×  *f*_1_ and 2 ×  *f*_2_) and the intermodulation frequency (*f*_1_ + *f*_2_) show a distinct increased amplitude. There are no differences in amplitudes between the two conditions, as such. **b** Time–Frequency Plot averaged across the indicated twenty occipital electrodes. Clear and long lasting SSVEPs are visible at driving frequencies and their harmonics, indicated by dotted horizontal lines

To validate that our design was suitable to elicit a robust SSVEP signal, we performed a fast Fourier transform across all participants and all electrodes at − 1000 to 2900 ms. The results, visualized in Fig. 2a, confirm that we succeeded in eliciting oscillatory brain responses at the driving stimuli’s frequencies, their harmonics, and the intermodulation frequency, that is, *f*_*1*_ + *f*_*2*_ = 20.57 Hz (note: the spectrogram is based on an average across all electrodes, thus no condition-related differences are to be expected).

### Data analysis

#### Behavioral data

Regarding the magenta dot detection task, the missing rates and the estimated detection rates d’ were calculated from hits and false alarms using the log-linear approach due to some hit rates = 1 and some false alarm rates = 0 (Hautus 1995). We conducted *t*-tests to determine whether participants directed equal attentional resources during both experimental conditions. We also performed a one-sample *t*-test to test against a chance performance which would yield *d*′ = 0.

#### SSVEPs in electrode space

We decomposed the event-related response by means of Morlet wavelet analysis (Bertrand and Pantev 1994) and calculated the spectral decompositions for 1–30 Hz frequency range (~ 15 cycles per wavelet).

Because spectral amplitudes decrease with increasing frequency, the frequency-transformed signal was normalized across conditions (related and unrelated) and across time, but separately for each participant, each electrode and each frequency. That is, within each participant, each electrode, and each frequency we concatenated the spectral power at each time bin and both conditions and z-transformed the resulting data series to a mean of 0 and a standard deviation of 1. The z-transformed data were baseline-corrected − 300 to 0 ms with respect to the object pair’s onset. The time by frequency plot (Fig. 2b), showing the baseline-corrected and transformed data, shows that we succeeded in specifically triggering activation of the brain at the original stimuli’s frequencies (8.57 and 12 Hz) and their harmonics. This activation was stable at around 800 ms and was long-lasting during the whole trial. This is in line with previous study results also averaging activation from 800 ms onwards (e.g., Martens et al. 2011). For further analyses, the brain’s averaged response at 800 to 1800 ms at the driving frequencies (averaged activation at 8.5 and 12 Hz) and at the intermodulation frequency (20.5 Hz) were analyzed.

**Fig. 3:**
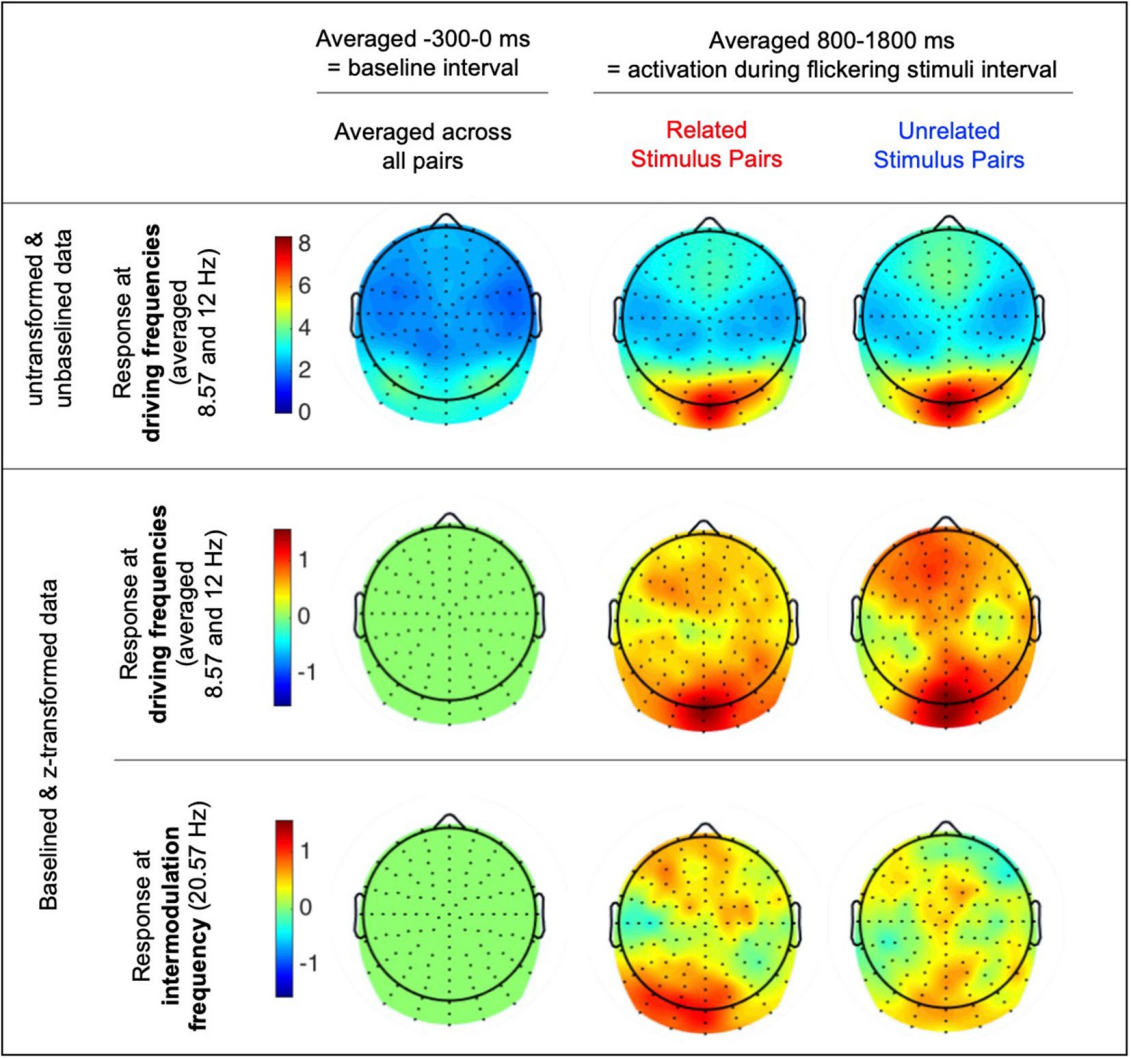
Topographic distribution of activity of untransformed and unbaselined data (at the top; to show the increase of occipial SSVEP with stimuli onset), for activity at the averaged driving frequency (middle) and at the intermodulation frequency (bottom) all at the baseline interval (left), and during flickering stimuli in the related (middle) and unrelated (right) condition

Figure 3 visualizes averaged activities during baseline (− 300 to 0 ms) and in the time range of interest (800–1800 ms)—each for activation at the driving frequencies and at the intermodulation frequency. The untransformed unbaselined data (first row in Fig. 3) shows that while the participants saw the paired flickering empty squares, oscillations built up at the driving frequency at typical occipital electrodes. This activation increased at the same occipital electrodes during the line drawing flickers. Therefore, the gray squares worked as SSVEP inducers and to control for perceptual features of the flickering stimuli and served as a perfect baseline in the following analysis.

To select suitable electrodes for further statistical analyses and to omit any selection bias, we opted for the following approach, similar to the approach in the seminal SSVEP experiment by Müller et al. (2003): We averaged the data from 800 to 1800 ms across related/unrelated stimulus pairs and across driving/intermodulation frequencies. For each participant, within the occipital 16 electrodes cluster marked in Fig. 2b, we selected the electrode with the maximum amplitude. We then took the four surrounding electrodes for each participant to create individual clusters of five electrodes for each participant. The averaged amplitudes at the selected occipital clusters were submitted to two *t*-tests. One *t*-test checked for amplitude differences in related versus unrelated stimulus pairs in driving frequencies and the other *t*-test checked the differences within the intermodulation frequency.

#### SSVEPs in source space

To localize the activation difference in intermodulation frequency between related and unrelated stimulus pairs, we used VARETA (Bosch-Bayard et al. 2001). This procedure provides the spatially smoothest intracranial distribution of current densities in source space, which is most compatible with the amplitude distribution in electrode space (Gruber et al. 2006). The inverse solution consisted of 3244 grid points (“voxels”) of a 3D grid (7 mm grid spacing). This grid and the arrangement of 128 electrodes were placed in registration with the average probabilistic MRI brain atlas (“average brain”) produced by the Montreal Neurological Institute (MNI; Evans et al. 1993). To localize the activation difference between related and unrelated stimulus pairs, a paired *t*-test with a significance level of *p* < 0.05 was performed. This procedure was used and described identically in other articles of our research group (e.g., Martens et al. 2011). Activation threshold corrections accounting for spatial dependencies between voxels were calculated by means of random field theory (RFT; Kilner et al. 2005; Worsley et al. 1996). The thresholds for all statistical parametric maps were set to *p* < 0.05. Finally, the significant voxels were projected to the cortical surface constructed on the basis of the MNI average brain. Area names for significant voxels were identified by the xjview toolbox (https://www.alivelearn.net/xjview) which uses the automated anatomical labeling toolbox (AAR2; Tzourio-Mazoyer et al. 2002).

## Results

### Behavioral

On average, participants missed 7.11% of the dots (*SD* = 5.47%). Importantly, missing rates did not differ between experimental conditions, *t*(16) = − 0.19, *p* = 0.85. Participant’s average detection performance of *d*′ = 4.09 (*SD* = 0.39), was significantly different from 0, indicating above chance detection rates, *t*(16) = 43.42, *p* < 0.001. Detection rates did not differ in trials with related versus unrelated stimulus pairs, *t*(16) = 0.35, *p* = 0.73. Therefore, participants attended equally well to all trials, irrespective of the experimental condition.

### SSVEPs in electrode space

**Fig. 4:**
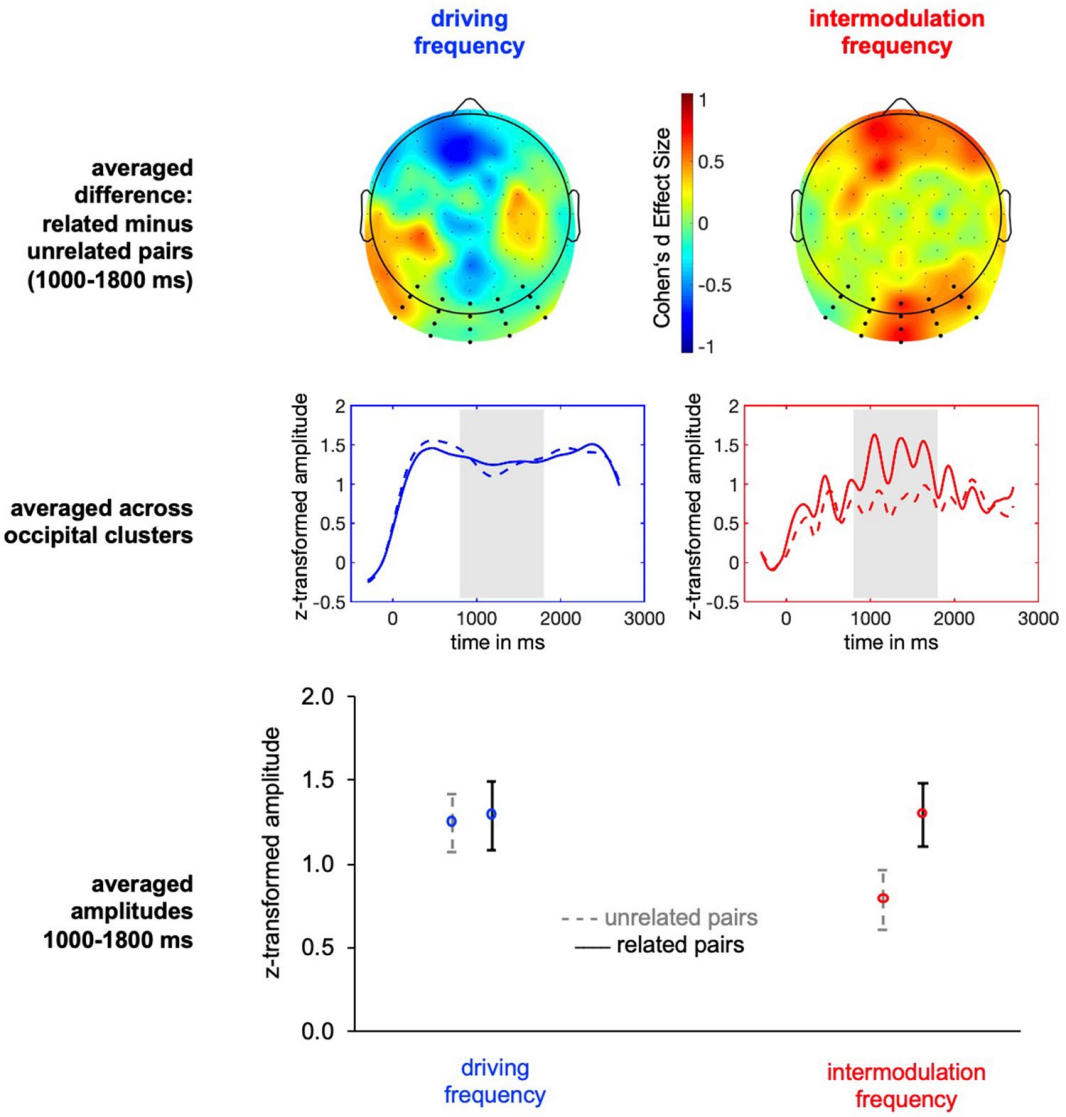
Top: Difference topographic distribution of activation during related minus unrelated stimulus pairs in driving versus intermodulation frequencies. Electrodes from which the individually maximally active electrode was chosen to form the five-electrodes cluster individually per participant are larger in size. Middle: Averaged amplitudes from the individual electrode clusters, averaged across participants. The shaded area (800–1800 ms) indicates the time of interest used for averaging among the time dimension. Bottom: Averaged activity 800–800 ms at the individually clustered electrodes. Error bars depict the confidence interval (95%)

The driving frequency amplitudes did not differ significantly when viewing related (*M* = 1.29, SD = 0.74) versus unrelated (*M* = 1.24, SD = 0.88) stimulus pairs, *t*(17) = 0.19, *p* = 0.853. The intermodulation frequency amplitudes were significantly increased when viewing related (*M* = 1.30, SD = 0.78) versus unrelated (*M* = 0.79, SD = 0.83) stimulus pairs, *t*(17) = 2.25, *p* = 0.038, Cohen’s *d* = 0.63. Effects and time courses are depicted in Fig. 4, separately for activation of driving (left) versus intermodulation frequency (right) amplitudes.

### SSVEPs in source space

**Fig. 5:**
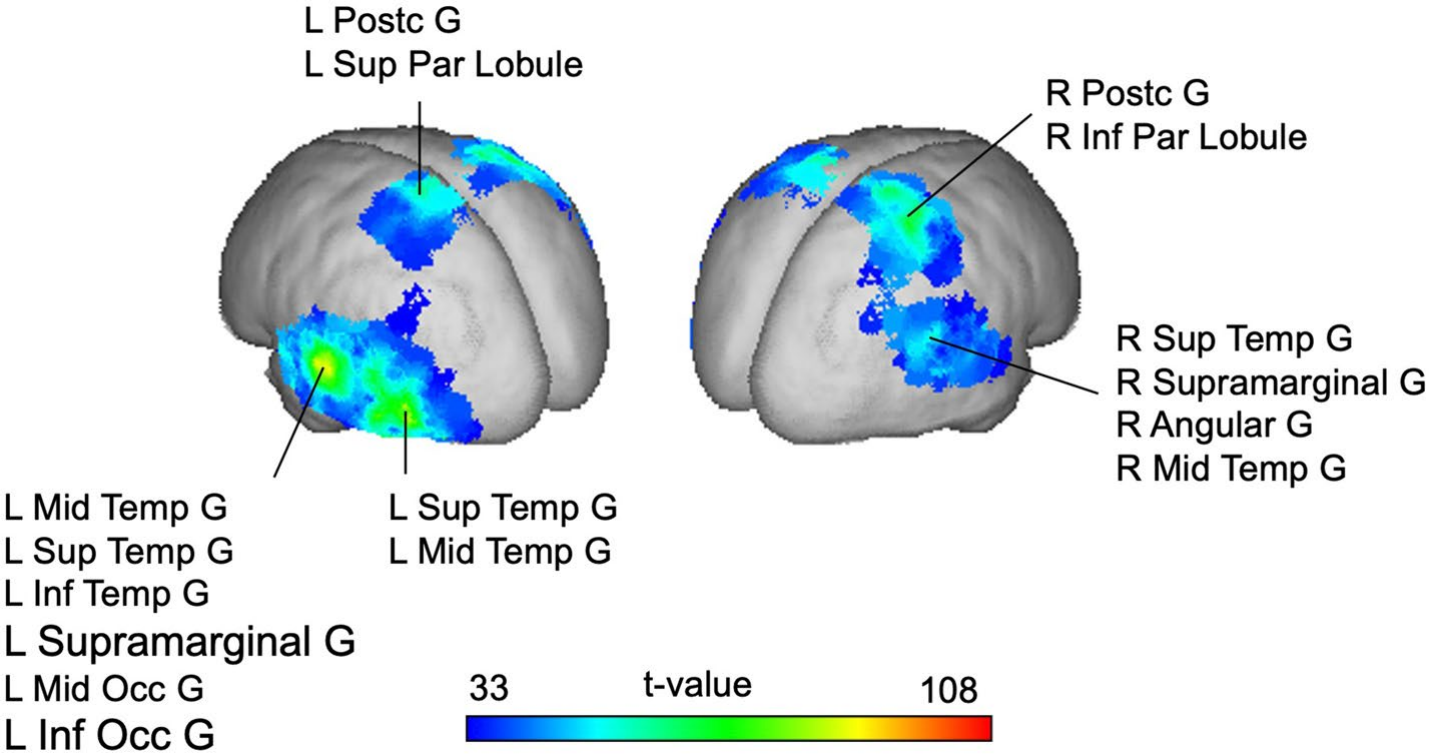
Difference activity (within intermodulation frequency: Related minus unrelated stimulus pairs): Statistically sigificant SSVEP Sources are marked, *p* < .05, RFT corrected. All regions with five or more significant grid points are labeled

The contrast of activity in the intermodulation frequency between related and unrelated stimulus pairs in the time window of 800–1800 ms after stimulus onset revealed significant effects in bilateral temporal and parietal areas. The centers of gravity of the sources revealing significant activation differences are specified in Table 2 and visualized in Fig. 5.

**Table 2:** MNI coordinates of the activation peaks

Cluster	Brain region	# Of grid points (total brain covers 3244)	MNI coordinates of local maxima		
			*x*	*y*	*z*
Left temporal (two local maxima)		138			
	L Mid Temp G	53			
	L Sup Temp G	36	− 57	− 33	12
	L Inf Temp G	13	− 50	− 62	− 10
	L Supramarginal G	9			
	L Mid Occ G	8			
	L Inf Occiput G	5			
	Undefined	5			
	areas with < 5 voxels each	9			
Right temporal		48			
	R Sup Temp G	25	36	− 33	63
	R Supramarginal G	9			
	R Angular G	8			
	R Mid Temp G	6			
Right parietal		43			
	R Post G	26	57	− 40	19
	R Inf Par Lobule	9			
	Areas with < 5 voxels each	8			
Left parietal		25			
	L Post G	15	− 21	40	70
	L Sup Par Lobule	8			
	Areas with < 5 voxels each	2

## Discussion

Using the SSVEP method, we investigated whether the intermodulation frequency can be used as an electrophysiological index for the integration of semantically related objects that are presented on a multi-stimulus display. Significant differences in the oscillatory brain response elicited by related versus unrelated objects evolved in the topographical and tomographical distribution of driving versus intermodulation frequencies. When viewing semantically related (versus unrelated) objects, the driving frequencies did not change in amplitude while the intermodulation frequency was associated with an amplitude increase. This amplitude change was associated with changes in specific temporal and parietal brain regions.

Previous studies showed that the intermodulation frequency is associated with a variety of functions that include neural interaction (for a review see Gordon et al. 2019). They are mainly involved in bottom-up processes, for example, low-level spatial interaction (Norcia et al. 2015; Victor and Conte 2000; Zemon and Ratliff 1984), attention (Kim et al. 2017) and perceptual binding (Aissani et al. 2011; Alp et al. 2016; Boremanse et al. 2013; Gundlach and Müller 2013). Only a very small number of studies hint at functions in high-level processing and the integrative function of intermodulation frequencies (Boremanse et al. 2014; Cunningham et al. 2017; Kim et al. 2017). Our results complement these findings.

Extracting the general meaning of a scene can occur due to a scenic context (= global gist) or due to semantic associations between simultaneously presented objects (= local gist) – where the latter is the focus of the present study. Local gist perception relies on the activation of local gist features and semantic memory. Intermodulation frequency might be generated in cortical areas activated by features that are driven by both frequencies (i.e., “local gist features”). However, the question persists where exactly in the brain these features are represented. According to the model by Bar et al. (2006), one might expect that local gist is triggered by orbitofrontal activity (Bar et al. 2006; Horr et al. 2014; Luu et al. 2010). This would indicate that local gist extraction is a similar process as global gist extraction. Alternatively, one might expect that the processing of local gist merely reflects interactions of local object representations, and, thus, activity centered around the lateral occipital complex should occur. In line with this second assumption, our source analysis revealed bilateral temporal and parietal lobe activations that are associated with the increase in intermodulation frequency amplitude when viewing related versus unrelated stimulus pairs. More specifically, neuroimaging studies showed that the inferior parietal and large parts of the middle and inferior temporal, lying at convergences of multiple perceptual processing streams, are involved in semantic processing (Binder and Desai 2011; Yee et al. 2018). Therefore, local gist perception in our study might be based on the interactions of local object representations which specifically support semantic memory processes.

It is unlikely that our findings can be attributed to the differences in different spatial frequencies within related and unrelated object pairs. It has been shown that top-down processing is involved in object recognition and scene perception, for example, low spatial frequencies from the image are rapidly projected to the PFC which in turn activate expectations and these initial guesses are back-projected to IT, where it is integrated with bottom-up processes (Bar 2003; Schöne et al. 2018). Because in our study, the line drawings in the two conditions had equal entropy of the spatial information and luminance, the effects are independent of these features. Furthermore, it is unlikely that our findings can be attributed to differences in attention. In all trials, participants had to detect a magenta dot and their reaction time did not differ when viewing related versus unrelated stimulus pairs. Therefore, the amount of attentional resources directed towards the stimulus pairs was not increased in the related compared to the unrelated pairs.

Recognizing the semantic relationship of two objects is achieved by linking component parts via associative relationships (Bar 2004) that are stored in declarative memory. Thus, local gist perception relies on the retrieval of this information. However, according to the dual-process model (Brown and Aggleton 2001; Donaldson 1996), information retrieval can take place via different processes. Both, familiarity, (i.e., the subjective feeling that an item has been experienced in the past) and recollection (i.e., conscious remembrance of prior events which also includes the retrieval of additional related information, for example, the circumstances during encoding) share similarities with gist perception, but it is unclear, which is the predominant retrieval process that underlies the rapid categorization of scenes.

In this study, we showed that SSVEPs are generally suitable for investigating gist perception with multi stimulus displays. However, future studies should investigate whether the choice of the specific driving frequencies and resulting intermodulation term affect the observed processes—we chose frequencies around 10 Hz, because the SSVEP signal is known to be largest around this frequency—however, this does not necessarily mean that this is the best frequency to observe gist perception processes.

It should be noted that the semantically related/unrelated objects were presented (1) without any background or context and (2) they were spatially well separated. Thus, it remains open to which extent the present findings can be transferred to settings that are more realistic.

Related to the first aspect, in the real world, the context is crucial for scene processing, specifically, the background-figure-interaction serves as a source of information and helps object recognition via contextual associations (Oliva and Torralba 2007). Using SSVEPs, Martens et al. (2011) investigated responses to scenes that consist of an object in the foreground and a landscape in the background. Similar to the present study, the object and landscape were presented at different driving frequencies and were either semantically related or unrelated. The findings by Martens et al. demonstrate that the impact of coherence on SSVEPs is not exclusively related to objects presented in isolation. It remains the challenge for future studies to examine the role of the intermodulation frequencies in such more realistic settings.

Regarding the second aspect, one could argue that the spatial separation of our two coherent objects limits the transferability of our results to real-world perception. Processing spatial relations between objects is a crucial part of contextual object processing, which is essential in real-world-processing (Oliva and Torralba 2007). A study by Bar and Ullman (1996) demonstrated that in multiple-object scenes, proper spatial relations (e.g., a foot below a hat, i.e., “realistic” spatial positions in relation to a person’s body) versus improper spatial relations (e.g., a head and a shoe next to each other) were associated with improved recognition performance in the first case. Thus, the spatial relation between objects is a relevant feature during scene processing and the spatial separation has to be considered as a limiting factor of our study. A future study examining distance and “realistic” positioning between objects in a parametric fashion has to tackle this limitation. Nonetheless, it has to be underlined that our design allows for a well-controlled examination of sematic relatedness independent of contextual processing and spatial distance.

Additionally, it remains the challenge for future studies to further specify the precise functional role of intermodulation frequencies. In particular, one should consider the following: Receptive field size increases with the hierarchy of brain areas. For example, receptive field size spans from approximately 1° in primary visual cortex (V1) to 2° to 25° in temporal occipital cortex and to 2.5–70° in inferotemporal cortex (Kay et al. 2013; Rousselet et al. 2004). Receptive fields of neurons that are responsible for object processing are therefore large enough to span at least parts of both objects that we presented in our study. Additionally, because receptive fields are overlapping (Wilson et al. 1983), our two objects might lie within an overlapping area of multiple neighboring receptive fields. Therefore, increased intermodulation frequency in our study might not only be due to processing semantic relatedness and gist perception exclusively but also due to processing the two objects within one receptive field or within an overlap of two receptive fields. However, the high level of visual processing during scene perception makes it experimentally very challenging to design an experiment in which the constituting elements of a scene are processed in clearly separable areas. An alternative approach might be to modulate the amount of relatedness instead of the spatial separation. To give an example, the morphology of the intermodulation frequency could be studied in scenarios where two separate objects are integrated within a more complex context (e.g., a background scene) that either facilitates or hinders the establishment of a semantic relation. To give an example, a “carrot” and a “top hat” might only trigger the semantic link “snowman” if presented within a winter landscape.

In summary, using the SSVEP method, we demonstrated that intermodulation frequencies are a marker for semantic integration of objects in a multi-stimulus display. The results of the source analysis suggest that the increase in intermodulation frequency amplitudes reflects parallel and feed-forward processing of related objects, which is necessary to establish the local gist experience.
